# Play partner preferences among groups of unfamiliar juvenile male rats

**DOI:** 10.1038/s41598-024-66988-w

**Published:** 2024-07-11

**Authors:** Jackson R. Ham, Sergio M. Pellis

**Affiliations:** Department of Neuroscience, University of Lethbridge, Alberta, Canada

**Keywords:** Social behaviour, Cooperation, Neuroscience

## Abstract

Like many mammals, as juveniles, rats engage in play fighting, which in the laboratory is typically studied in dyads, and consequently, it is the researcher who determines a rat’s play partner. In real-life conditions, a rat would have many partners with whom to play. In a previous study, we found that rats do prefer to play with some individuals more than others, and surprisingly, when given the choice, unfamiliar partners are preferred to familiar ones. In this study, we assessed partner choice when all the available partners are strangers. Eight groups of six unfamiliar juvenile male rats were observed for 10 min play trials. One of the six in each group was selected as the ‘focal’ rat and his play towards, and received by, the others were scored. Social networks revealed that five of the eight groups formed preferences, with preferred partners also engaging in more play with the focal rat. The mechanism by which these preferences were formed remains to be determined, but it seems that there are individual differences, potentially in the amount and style of play, that allow an individual to select the most suitable partner from a group of strangers.

## Introduction

Play fighting or rough-and-tumble play is one of the most commonly reported forms of play behavior^1^, and is observed in many mammals, especially as juveniles^2^. The defining feature of play fighting is that animals compete for an advantage, which, in many cases, is to contact a particular location on the partner’s body, but do so while showing some degree of cooperation^3^. The advantage/targets vary across species and lineages of species^4^, being derived from targets typically bitten, rubbed, or otherwise contacted during such adult functional contexts as conspecific aggression, predation, sex, and other forms of affiliative behavior (e.g., grooming)^5^. The cooperation present in play fighting leads to reciprocity and turn taking, ensuring that the encounters remain playful^6,7^.

Play fighting has been most intensively studied in laboratory rats (*Rattus norvegicus*)^8,9^, in which the animals compete to nuzzle each other’s napes with the tip of the snout^10,11^. Rats are highly motivated to play^12^, and play engages neural circuits spanning the entire neuraxis^13–15^. The play fighting experienced in the juvenile period influences the development of socio-cognitive skills and does so by altering the anatomy and physiology of the prefrontal cortex^9,16,17^, with similar findings also reported in two other species of rodents^18,19^. In rats, play fighting continues into adulthood, albeit at a lower frequency^20,21^, and is used to assess and manage social relationships, especially dominance relationships^22,23^. Both the juvenile function of play fighting as a tool for refining socio-cognitive skills, and its use as a tool for social assessment and management in sexually mature animals, may extend beyond rats and some other rodents to include mammals from many other lineages^24–26^.

Typically, play in laboratory rats is evaluated with the ‘dyadic test,’ in which two animals are placed in a neutral arena after a period of social isolation, which can range from a few hours to several days^27^. Play trials last from 5 to 30 min, with playful attacks to the nape beginning within the first 1–2 min^28^. The number of attacks to the nape is an effective measurement to assess the motivation to play^27,29,30^.

While the ‘dyadic test’ has been a useful tool for studying play in rats^27^, it has a limitation. In the wild, rats live in colonies, with litters from multiple females born in close temporal proximity^31^, affording juveniles the opportunity to play with many other juveniles, both siblings and non-siblings. Thus, unlike in the dyadic test, in more natural settings, rats have a choice of play partners.

Other species reared with littermates or in social groups with multiple similar aged partners exhibit preferences in their selection of play partners^32–34^. Similarly, when living in groups, rats prefer to play with some members more than others^9,27,35^. Even in the dyadic test, not all partners presented seem equally attractive, with how much play directed at the partner being dependent on their sex, relative difference in age, whether they are of the same or different strain, and whether they are familiar or novel partners^20,36–39^. This suggests that, when given the choice among different potential partners, rats will show preferences.

In a recent study, we evaluated one factor that seems likely to be important in selecting potential partners: familiarity^40^. In this study, rats were tested in groups of four, with the focal rats being given a choice of three partners. One partner was a cage mate (i.e., familiar), another was housed on the other side of a transparent and perforated divider (i.e., somewhat familiar), and the third was a stranger from a separate cage (i.e., unfamiliar). The focal rats initiated playful nape attacks differentially: familiar < unfamiliar < somewhat familiar. Focal animals did not vary their play style with animals of varied familiarity, only the amount of play was altered. Moreover, whereas the focal rats did not alter the amount of play they initiated with familiar and somewhat familiar partners with the magnitude of difference in weight and dominance, they did so with fully unfamiliar partners, initiating less play with heavier, more dominant animals^40^. Given that within a group of familiar rats there are partner preferences^9,27^, and that when given a choice rats prefer to play with less familiar partners^40^, in this experiment we tested whether within a group in which all the rats are strangers, they would form partner preferences.

Play fighting in the juvenile period influences the development of prefrontal neural connections^41^ that are important for both coordinating motor actions with a partner and adjusting how they play depending on the identity of the partner^42^. The former depends on the quality of the play^43,44^ and the latter on the diversity of partners to which the rat is exposed^41^. Therefore, knowing if rats form preferences with unfamiliar rats during a play session has implications on whether juvenile rats choose to socialize and play with partners that maximize the relevant experiences that lead to these socio-cognitive improvements. That is, young rats may actively create a social niche that is functionally beneficial to them^45^.

In the present study, groups of unfamiliar male juvenile rats were tested together to determine if rats form partner preferences in play and if they do, what factors they base these preferences on. One rat was selected as the focal animal and its partner preferences was assessed by the relative proportion of nape attacks directed to different members of the group^9^. We predicted that rats would form preferences when playing with unfamiliar partners. After establishing whether or not focal rats had partner preferences, several possible factors that could influence the emergence of those preferences were explored. Rats have individual differences in how much they play^46,47^, but will modify the expression of that playfulness based on the playfulness of their partner^30^. Therefore, we predicted group mates that initiated more nape attacks towards the focal rats would be preferred by the focal animal. Rats that play more also tend to prefer to use different defense tactics than rats that play less^27^. Therefore, we predicted the relative difference in types of defenses used between preferred and non-preferred partners that reflect differences in the degree of body contact during play would influence partner preferences. In addition, a measure reflecting the degree of cooperation was compared on the assumption that since pairs with more symmetry are more likely to gain the experiences most beneficial for the development of inter-animal coordination skills^39,48,49^, rats would select to play with more compatible partners. Given that differences in weight and dominance influenced the amount of play directed towards strangers in another group play experiment^40^, weight and dominance differences were compared between the most and least preferred partners. In addition to play style, weight, and differences in dominance, rats may select partners based on proximity. That is, rats may pick to play with the individuals that are closest to them. To determine if social proximity influenced partner selection, we measured the time individuals spent close to one another. Finally, the temporal distribution of playful attacks was scored to determine when in the trial preferences were formed or consolidated^40^. In this way, not only was the existence of partner preferences assessed, but also some of the possible factors contributing to their formation were tested.

## Methods

### Ethics statement

All care and testing procedures were reviewed and approved by the University of Lethbridge Animal Welfare Committee (protocol #1809) in compliance with guidelines from the Canadian Council for Animal Care and comply with the ARRIVE guidelines.

### Subjects

Forty-eight weanling Long Evans male rats were purchased from Charles River Laboratories (Kingston, NY, USA) and arrived at the Canadian Centre for Behavioural Neuroscience at 22 days of age. Upon arrival, the animals were moved into double decker cages with corncob bedding and housed in groups of 6 (resulting in 8 groups). Food and water were available ad libitum. Animals were housed on a 12-h light–dark cycle with lights off at 19:30 and maintained at a constant temperature of 21–23 °C.

### Apparatus

Animals were tested in a large Plexiglas® enclosure (80 cm × 80 cm × 50 cm), that was specifically scaled up from our enclosure that is used to test dyads, to accommodate groups of six rats. Corncob bedding was used to sufficiently cover the floor of the enclosure. An ExmourRS 4K Sony Handycam was used for filming the play sessions and was place over top of the enclosure at nearly a 90° angle.

### Procedure

At 28 days of age, the rats were habituated to the test enclosure for 10 min, with their cage mates, in red light. This was done for two consecutive days between 07:30 and 19:00. At 30 days of age, the rats were sufficiently habituated to the enclosure and testing began. Groups of cage mates were placed in the enclosure, in red light, for 20 min and filmed. The play trials were repeated for eight days. After testing, animals were rehoused with their cage mates for 24 h. Before each play trial, the rats were socially isolated for 2.5 h to increase their playfulness^27^. Before being socially isolated, each rat was weighed. The bedding was replaced, and the test enclosure was cleaned with Virkon® after each trial to reduce any odors left from the previous rats. To identify individual rats, the tail was colored, using different patterns, with a permanent marker pen (Sharpie®). After eight days of testing animals with familiar cage mates, they were tested in two consecutive play trials with unfamiliar animals, when they were 38 and 39 days old. These groupings were assigned randomly so that all the rats in each group were unfamiliar with each other. Even when seemingly redundant, such test trials were run twice because of the risk of data loss due to instrument failure or some unexpected event, such as load noise in another part of the laboratory, that suppresses play in the rats^27^. As all test trials from the first session when the rats were 38 days old worked, data from the first trial with unfamiliar groups were analyzed for the present study.

Following each play trial, the tube test^50^ was employed to determine the relative dominance among members of each group tested. To do so, pairs of rats from the play group were placed headfirst into a Plexiglas® tube (19.5 cm in length and 4.5 cm in diameter), simultaneously, at opposing ends. The tube was just large enough to allow one rat through, but not so large that the second rat could squeeze past its opponent. The ‘loser’ or subordinate rat was determined as the rat that was pushed out of the tube, while the ‘winner’ remained in the tube. The winning rat was given a point for that round. If neither rat was pushed out after 60 s, the round was scored as a tie, and no point was awarded for that encounter. Each possible pairing within the group was tested five times and the sum of points was used to determine which was the most dominant. After five rounds, the testing for any given pair was completed, the tube was cleaned with Virkon®, and the next pair was tested.

### Behavioral analysis of play trials

The first 10 min of each video recording was analyzed using both normal speed and frame-by-frame analysis to score various aspects of the rats’ playful attack and defense strategies^27,29^. Only the first 10 min was used as the majority of play, following brief social isolation, occurs in the first 10 min after partners have been introduced to one another^40,51^. For each video, one of the six rats was selected to be the focal animal. For group one, rat one was selected, for group two, rat two was selected, so on and so forth until the seventh and eighth group in which rat one and rat two were selected as the focal animal, respectively. Once selected, we used a focal follow scoring approach. Both the attacks initiated and directed toward the focal animal were recorded, with the individuals attacking and defending identified and recorded by their tail markings. A playful attack was scored when the snout of a rat made contact with the nape of another rat as this is the target in rat rough-and-tumble play in around 90% of playful attacks^29^. Additionally, if a playful attack was initiated and directed towards the nape, but the defender evaded before contact could be made, this too was scored as a playful attack. How the rats responded to a playful attack was scored following the protocol described by Pellis et al.^27^ and is summarized in Table 1. The number of playful nape attacks were scored as the number by each animal per trial, whereas how rats responded to attacks were scored as a percentage of the attacks. Thus, defense was calculated the percentage of attacks defended compared to overall attacks, which is a measure of how motivated the recipients are to engage with the attacker. Of the defended attacks, the percentage leading to evasive defense was used as a measure of how reluctant the recipient was in engaging in close quarter wrestling. Similarly, of the defended attacks, the percentage that led to pinning is a measure of the animal’s motivation to sustain close bodily contact when playing^39^. The percentage of defended attacks that lead to role reversals is a measure of both reciprocity and the motivation to continue the ongoing interaction. The relative symmetry in initiating role reversals has been found to be a good measure of coordination between play partners^38,39^. This is a measure of the pair’s behavior, calculated as the number of role reversals initiated by rat A/total role reversals (those by A + B) (Table 1). Converted to a percentage, the resultant value can be interpreted as the closer to zero, the more mutual the interaction, and the closer to 100%, the more asymmetrical the contribution by the partners^39^.

**Table 1:** The behavioral measures used in this study.

Behavior	Definition	Calculation
**Attack**		
Nape attack	Playful contact made with the snout on the nape of a partner	∑ Frequency with each partner = total play
**Defense**		
% Defended	Playful attacks that are responded to with either an evasion or a facing defense	% defended = [(Total attacks launched to a given partner – occurrence when the animal does not respond) / total attacks launched to given partner] × 100
% Evasion	Partner runs away after being attacked	% evasion = [(frequency of defended attacks – frequency of attacks evaded) / frequency of defended attacks] × 100
% Pin	Partner defends nape by rolling into a supine position	% pin = [(frequency of defence attacks – frequency of attacks resulting in a pin) / frequency of defended attacks] × 100
**Role reversals**	When the role of the defending rat switches to that of attacker (i.e., turn taking)	% role reversal = [(frequency of defended attacks − frequency of role reversals)/frequency of defended attacks] × 100
**Asymmetry in role reversals**	The degree to which turn taking is shared between a pair. A number closer to 0% indicates that for every role reversal Rat A engages in Rat B engages in a similar amount. If closer to 100%, this indicates one of the rats is engaging in all of the role reversals	$\text{Asymmetry}=\left(\frac{|\left(\text{Rat A role reversals}\right)-\left(\text{Rat B role reversals}\right)|}{\sum \text{Rat A }\rightleftharpoons \text{Rat B role reversals}}\right)\times 100$
**Social proximity**	Time spent within one body length of partner	Social proximity = total duration focal and Rat A are within one body length of each other − (time focal spent playing with Rat A + time Rat A spent playing with the focal rat)

Finally, the duration of time spent in close social proximity was measured. This was done to determine whether the focal rat played with a partner that was closest and most convenient or instead if they seek out a particular partner within the play box. Rats were considered to be in social proximity if they were within one body length of each other^40^. As some of the time spent in close social proximity was due to the pair playing, the total time the two individuals spent playing with each other was subtracted from the total time they were in proximity to each other^40^. All behaviors measured are summarized in Table 1.

### Statistical analysis

#### Partner preferences

To visualize partner preferences, egocentric networks were plotted using *igraph*^52^ in R^53^. The central node, or central circle, represents the focal animal in each group, with the surrounding nodes representing the potential play partners. The edges, or lines, radiating from the central node are unidirectional and the thickness visualizes the proportion of play the focal animal initiated (N.B., these edges only show focal initiated play and not how much the partners played with the focal rat). The proportion of play, rather than frequency, was calculated and used to make comparisons as there is intra- and intergroup variation in the amount of play initiated by individuals. The proportion of play was calculated by dividing the number of playful attacks directed to a given partner by the focal rat by the total number of playful attacks launched by the focal. To determine if focal rats preferred individuals within their group significantly more than others, Chi-square goodness of fit tests, using the raw scores play scores for each focal rat, were used to determine if the distribution of nape attacks among the five partners was different from what would be expected if the nape attacks were distributed evenly (i.e., 20% of the focal rats play per partner) for each focal rat. If the Chi-square tests revealed significant deviations from what would be expected by chance, further tests on the other behavioral parameters scored were used to determine what may be driving partner preferences.

#### Potential factors influencing partner preferences

While measures of the microstructure of play fighting rats have improved in the past decades^27^, they are still too crude to ensure that subtle differences in style are detected. Sometimes, it is the reluctance of the rats to engage with some partners that provide indirect clues that there is something different in how a specific rat is playing^49^. Therefore, once the Chi-square test revealed a significant difference in how much play was directed to and from potential partners, trials from the most extreme differences—the most and the least preferred partners—were analyzed using Wilcoxon ranked sign tests in SPSS (Version 29). This analysis included the number of nape attacks launched by the focal rat’s partners, preferred tactics of defense, weight asymmetry, dominance asymmetry, social proximity, and latency to first attack by the focal rat to the most and least favorite partner. If there was more than one most or least favorite partner for a focal rat, the measures were averaged. Focal rats that did not express partner preferences were excluded from further analyses.

As rats show a great deal of individual variation in the initiation of playful attacks, a Mann–Whitney U test was used to determine if rats that form preferences initiate more playful attacks than those who do not form preferences. Finally, the temporal distribution of playful attacks directed to each of the five potential partners, over the 10 min play sessions, initiated by the focal rat, was plotted using the *ggplot2* package^54^.

## Results

### Partner preferences

**Figure 1:**
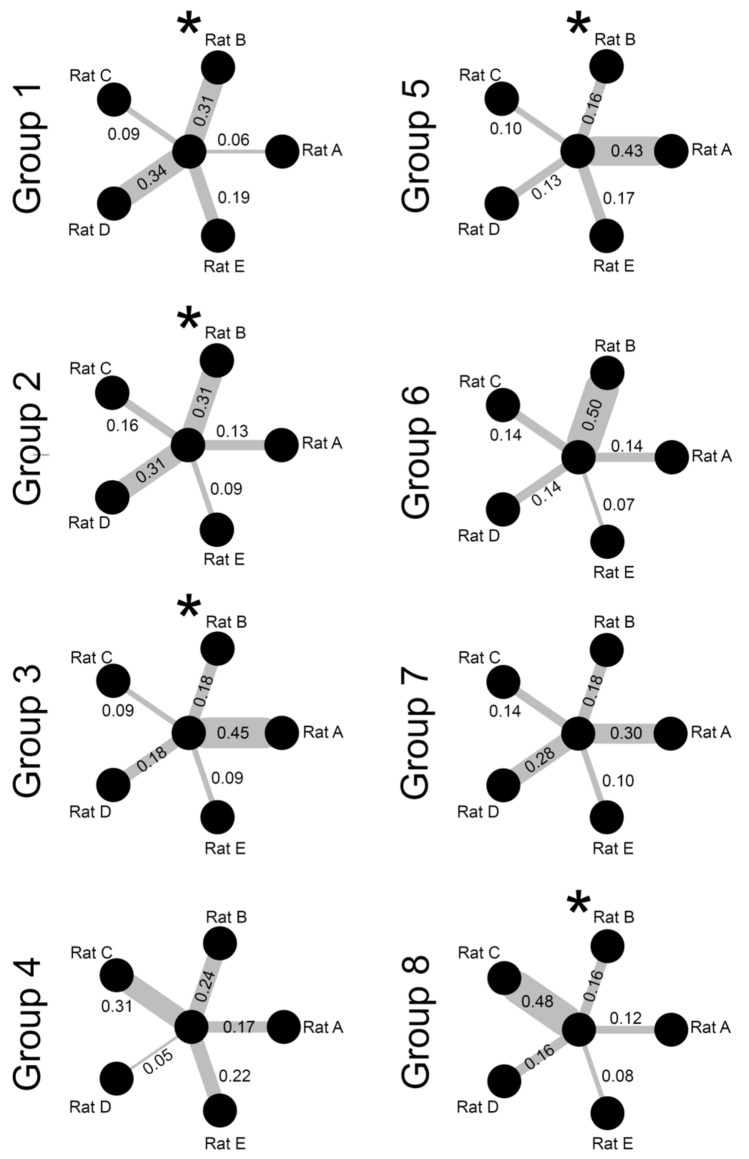
Egocentric social networks are plotted showing the proportion of play focal individuals (center node) directed towards the five other rats in the group. Individual networks are plotted for all eight focal rats, with an asterisk indicating which focal animals differed significantly from a chance distribution (see Table 2 for statistical analyses). *  Indicate when a focal rat had significant preferences.

Egocentric social networks plotting the proportion of nape attacks directed to each of the five play partners showed that all eight focal rats formed preferences (Fig. 1). To determine if these preferences were significant, Chi-square tests were used to evaluate whether the playful attacks directed to each partner differed significantly from what you would expect if each partner was attacked equally, based on chance (20%). The Chi-square tests revealed that five of the eight focal rats formed partner preferences (Table 2).

**Table 2:** The number of nape attacks expected, by chance (20%), based on the total number of nape attacks launched is tested against the number of nape attacks actually launched to each partner.

Focal		Rat A	Rat B	Rat C	Rat D	Rat E	Total	Chi^2^ test
1	Nape attacks	2	10	3	11	6	32	***X***^**2**^** = 10.19, df = 4 *p* = 0.037**
	*Expected*	*6.4*	*6.4*	*6.4*	*6.4*	*6.4*		
2	Nape attacks	7	14	6	14	4	45	***X***^**2**^** = 9.78, df = 4, *p* = 0.044**
	*Expected*	*9*	*9*	*9*	*9*	*9*		
3	Nape attacks	10	4	2	4	2	22	***X***^**2**^** = 9.82, df = 4, *p* = 0.044**
	*Expected*	*4.4*	*4.4*	*4.4*	*4.4*	*4.4*		
4	Nape attacks	7	10	13	2	9	41	*X*^2^ = 8.15, df = 4 *p* = 0.086
	*Expected*	*8.2*	*8.2*	*8.2*	*8.2*	*8.2*		
5	Nape attacks	13	5	3	4	5	30	***X***^**2**^** = 10.67, df = 4 *p* = 0.031**
	*Expected*	*6*	*6*	*6*	*6*	*6*		
6	Nape attacks	2	7	2	2	1	14	*X*^2^ = 8.14, df = 4 *p* = 0.086
	*Expected*	*2.8*	*2.8*	*2.8*	*2.8*	*2.8*		
7	Nape attacks	15	9	7	14	5	50	*X*^2^ = 7.60, df = 4 *p* = 0.11
	*Expected*	*10*	*10*	*10*	*10*	*10*		
8	Nape attacks	3	4	12	4	2	25	***X***^**2**^** = 12.80, df = 4 *p* = 0.012**
	*Expected*	*5*	*5*	*5*	*5*	*5*		

Significant values are in bold.

### Potential factors influencing partner preferences

When comparing the most favorite and least favorite partners with Wilcoxon ranked sign tests, we found that the only significant difference was in the amount of play the partner initiated, with favorite partners directing more play towards the focal rat than the least favorite partner (Table 3). Weight and dominance asymmetries did not influence preferences, nor did the time spent in social proximity or latency to first attack.

**Table 3:** Comparisons form the most extreme differences. Only play from focal rats that had significant preferences (i.e., Focal 1–3, 5, and 8) are compared. Significant values are in bold.

Behavior	Most preferred (mean ± SD)	Least preferred (mean ± SD)	Wilcoxon ranked sign test
Nape attacks towards focal	8.0 ± 2.92	4.9 ± 2.75	**Z = 15, *p* = 0.042**
Weight asymmetry (g)	11.99 ± 24.90	3.94 ± 22.37	Z = 6, *p* = 0.69
Dominance asymmetry	− 2.6 ± 10.71	− 1.7 ± 9.58	Z = 8, *p* = 0.89
Social proximity (s)	145.54 ± 19.36	175.91 ± 21.63	Z = 13, *p* = 0.14
Latency (s)	45.35 ± 23.39	66.27 ± 40.45	Z = 12, *p* = 0.23

Wilcoxon signed-rank tests comparing the difference in how the focal rats who formed preferences responded to attacks by the most preferred and least preferred partners, and how these partners responded to the focal rats, revealed no significant differences (Table 4). Neither focal rats, nor their partners, differed significantly in the likelihood of defending themselves, using evasive defense tactics, ending in a pin configuration, performing role reversals, or in the symmetry of role reversals.

**Table 4:** Comparisons from the most extreme differences—the most and least preferred partners—are presented below to compare various measures of their play behavior.

Behavior	Most preferred (% ± SD)	Least preferred (% ± SD)	Wilcoxon ranked sign test
% Defense			
Focal	80.49 ± 13.13	61.67 ± 26.09	Z = 4, *p* = 0.35
Partner	66.29 ± 29.59	63.75 ± 37.72	Z = 6, *p* = 0.72
% Evasion			
Focal	42.60 ± 16.41	50.00 ± 50.00	Z = 9, *p* = 0.69
Partner	59.39 ± 27.07	56.19 ± 34.94	Z = 4, *p* = 0.72
% Pin			
Focal	33.49 ± 14.90	21.67 ± 21.73	Z = 1, *p* = 0.08
Partner	26.35 ± 26.71	9.52 ± 14.68	Z = 2, *p* = 0.27
% Role reversal			
Focal	29.19 ± 11.69	25.00 ± 43.30	Z = 5, *p* = 0.50
Partner	49.44 ± 26.08	2.86 ± 6.39	Z = 0, *p* = 0.07
% Asymmetry in role reversals	36.67 ± 36.36	79.36 ± 43.41	Z = 9, *p* = 0.14

**Figure 2:**
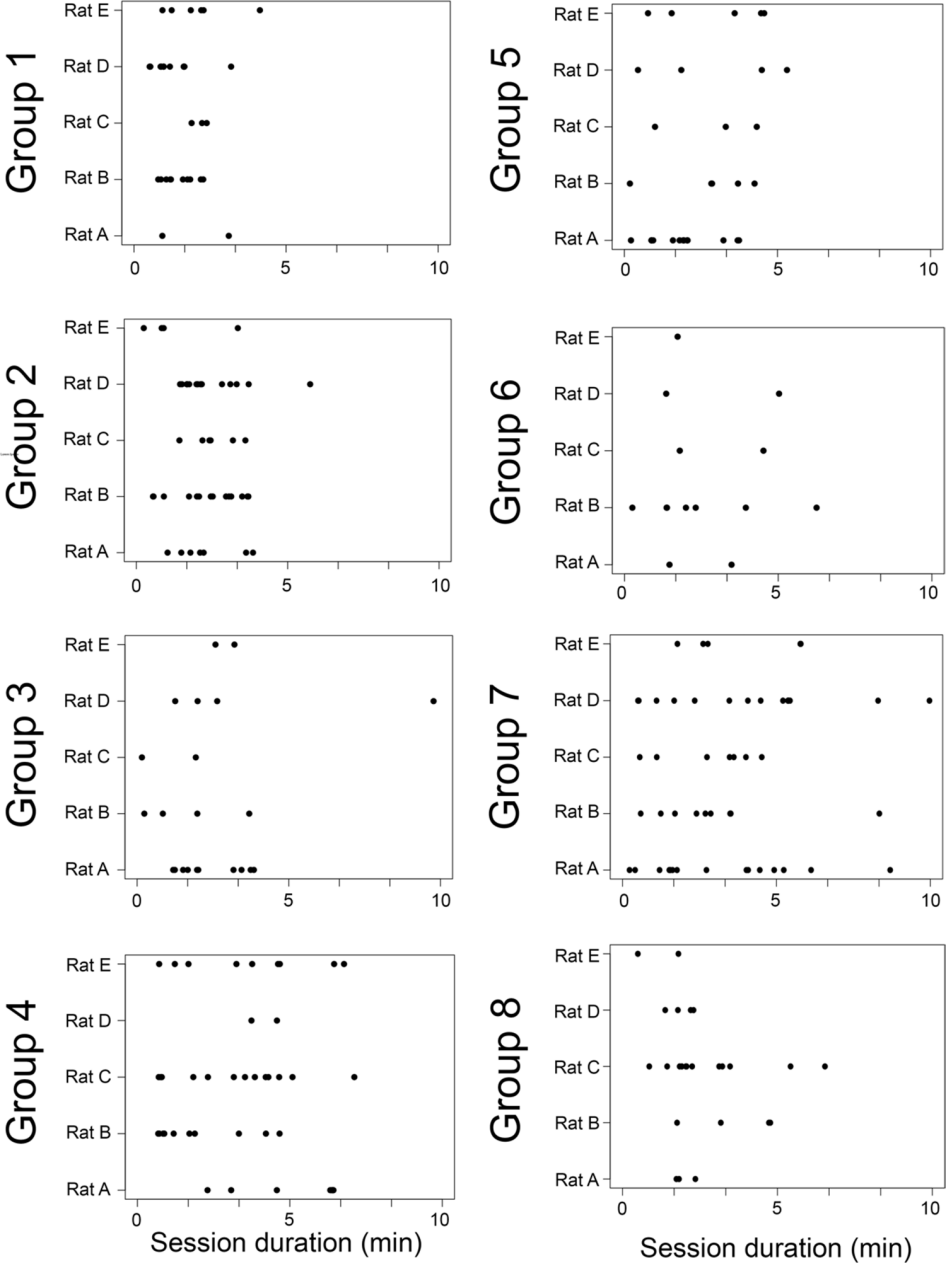
The temporal distribution of playful attacks across the 10 min session is plotted, illustrating when the focal rat-initiated play with each of the five partners, for all eight focal rats from each group.

The temporal profile of attacks by focal rats on each of their partners over the test period revealed there were marked individual differences in the amount of play initiated by each of the eight focal rats (Fig. 2), but high and low playing focal rats did not differ in the expression of their preferences. Moreover, when comparing the number of nape attacks initiated by focal rats that form preferences (30.0 ± 8.87) against those that do not (41.0 ± 18.73), a Mann–Whitney U test revealed that there was no difference in playful initiation (*z* =  − 0.27, *p* = 0.79).

## Discussion

Within groups of rats that are familiar with one another, data indicate that they have partner preferences^5,27^, and when confronted with familiar and unfamiliar partners, rats have a preference to engage unfamiliar rats in play^40^. In the present study, even though the groups of six rats had never encountered one another before, five of the eight selected focal rats exhibited partner preferences (Fig. 1), significantly directing more play to some members of the group than others (Table 2). Importantly, these preferences were not an artefact of playing with the nearest available partner. The overall amount of time spent in close proximity when not playing did not account for why one partner was preferred over another. Indeed, a focal rat would leave a rat it was next to and travel to a distant location to launch a playful attack on another rat. So, even though not all focal rats show significant partner preferences, in those that did, the choice involved was an active one.

Of the focal rats with preferences, the preferred partners also directed more playful attacks to the focal rats (Table 3). These findings are surprising as the groups consisted of same strain, same sex, same age strangers, and the results presented are based on them interacting for only 10 min, so providing little time and no obvious features for individuals to distinguish quickly between more preferred and less preferred play partners.

Despite five of the focal rats showing preferences for certain partners, three did not. This suggests that while some rats are more sensitive to the partners with which they play, others may be more gregarious and so less choosy about play partners. Though it is typical to socially isolate rats before testing play^27^, perhaps the time spent in isolation before testing influenced whether rats formed preferences. By isolating the rats, the individuals are known to have not played before a play session and thus, should be motivated to play. However, this motivation to play may have influenced the results of this study. If the focal rats are highly motivated to play, due to the social isolation, it may mask preferences, as the rewards of playing may outweigh the motivation to find a suitable play partner. Indeed, even among the focal rats that formed preferences, it remains unknown if these preferences would be even stronger had they not been isolated. Differences in isolation time and its’ influence on partner preferences should be investigated in the future.

Nonetheless, given that partner preferences can be established in such situations by some rats, this has a couple of major implications. First, it provides evidence that forming partner preferences may be an important means by which young rats establish a social milieu in whatever context they find themselves that give them the experiences they find most rewarding. That is, they create their own social niche^45^. Second, it could explain why there are typically such large variances in scores of play in the dyadic test, even when same sex, same condition rats from inbred strains of rats are used^30^. The random pairing of rats, which are artificially selected by experimenters, may contain preferred and non-preferred partners in the dyads, resulting in uncontrolled influences on both how much and how rats play. What remains to be determined is how rats confronted with multiple unfamiliar partners can so quickly identify which members of the group are preferred.

We assessed some intuitively obvious factors involved in partner selection for the focal rats that formed preferences. First, within established groups of juvenile male rats, the rats that become overtly identifiable as being dominant after sexual maturity tend to receive more playful attacks from the rats that when sexually mature exhibit clear signs of being subordinate, and this tends to be correlated with weight as the to-be dominants, on average, tend to weigh more^55^. Similarly, in trials in which the focal animal could choose between familiar and unfamiliar partners with whom to play, for strangers, preference was given to those which were less dominant and lighter^40^. That is, in some situations, the dominance of the potential partner, even among strangers^23^, can influence whether rats play with them and how they play with them. Again, surprisingly, when all potential partners were strangers, differential dominance relationships, as measured by the tube test, or differences in body weight, seemed to have no influence on which partners became the most and the least preferred play partners.

Second, there are individual differences in how playful rats are, with some consistently launching more nape attacks than others^30,46,47^, with pairs of high playing versus low playing rats tending to differ in some of the play tactics they predominantly use^9^. Therefore, we predicted that individuals within groups of unfamiliar rats would likely gravitate to rats that play similarly. Focal rats did differ in how much play they initiated (Fig. 2), but this had no impact on whether partner preferences were formed. In addition to the amount of play initiated, the style and quality of play was evaluated. Neither the focal rats’ play style nor the partner’s play style significantly influenced the selection of partners. Additionally, the quality of play, as measured by role reversals, play symmetry, and symmetry in role reversals, did not differ between most and least favorite partners.

Over the course of the 10-min trial, the focal rat continued to interact sporadically with all five potential partners (Fig. 2) rather than sampling each partner in the first minute and honing in on the preferred individual(s). Therefore, preferences could only be determined from the cumulative effect of counting all the nape attacks over the entire trial. The same pattern was also seen in the experiment on partner choice when confronted with rats of varying familiarity^40^. This suggests that, although some partners are preferred, that preference is not exclusive, with even the least preferred partner occasionally being attacked, even towards the end of the trial. Clearly, there is still much to learn about the cues that rats use in choosing which potential partners are preferred—if they form partner preferences—and why they continue to play with all partners, even the least preferred ones when preferred partners are available.

While animals often prefer to play with familiar individuals^56–58^, and this includes humans^59,60^, juvenile male rats generally prefer to play with individuals that are novel, but not completely novel^40^. Indeed, rats also choose to interact with novel partners over familiar partners in non-playful contexts^61^. Although the salient feature varies from species to species, several factors have been identified as influencing partner preferences in social play. Typically, animals prefer to play with individuals of similar age and of the same sex^62,63^. However, for some animals, these preferences change with age^32,33^ or depending on the type of social play performed^64^. In addition to preferring animals of the same sex and of a similar age, relative dominance status can also influence play partner preferences, with animals tending to prefer playing with partners of a similar rank^40,65,66^, although in some situations, rats do the opposite^55^. As partners of varying age and sex may provide different experiences (i.e., an older partner, who is more skilled, maybe a more challenging play partner), understanding which partners are preferred, for a given species, and how these preferences are formed is important as they may reflect differences in the play experiences gained by juveniles.

For rats, it is still unclear what makes a partner ‘desirable’ and why some rats form preferences and others do not. However, partner choice is context dependent. For example, if preferences are only assessed through dyadic encounters, whereby you allow a focal animal to play with an unfamiliar one day and the next with a familiar animal, you are confounding your results as the rat is only afforded one choice in any given trial. That is, if the rat is motivated to play, it may inflate how much it plays with the partner if that is the only option. Additionally, the partner may alter how much the focal animal plays, with high-playing rats decreasing the amount of play they initiate if they are partnered with a high-playing partner^30^. However, it is still unknown if individuals prefer to play with complementary players (i.e., high players preferring high players, and low players preferring low players)^46^. Despite this inflation, dyadic encounters have found that individuals tend to play more with unfamiliar than with familiar animals; however, when rats are given the choice between familiar animals, somewhat familiar animals, and unfamiliar animals, they prefer somewhat familiar animals over strangers and familiar animals, but strangers over familiar animals^40^.

Comparing the results of this study to a previous study from our lab^40^ shows yet another example of how preferences and the mechanisms by which they are determined are context dependent. Although the rats had a choice between three individuals of varying familiarity, focal rat partner selection was influenced by relative weight and dominance asymmetry, at least when playing with strangers. However, focal rats in groups of unfamiliar animals are not influenced by differences in relative weight and dominance (present study). Although, it should be noted than in both studies dominance was measured using the tube test, which may not accurately reflect dominance and instead indicate differences in behavioral strategies. Consequently, the role of dominance will need further testing to be certain in how it may or may not influence play partner preference. Even so, the contrasting findings from these two studies suggest that the salient information used when making decisions on choice of play partners may change when the context changes.

Social play has been linked to the development of socio-cognitive skills in rats and hamsters^16,18,43,48^ and temperament refinement and reproductive success in ground squirrels^67–69^. Indeed, the quality of juvenile play experiences in rats significantly alters the development of the medial prefrontal cortex and social skills. It is not the amount of play but instead, the turn-taking or role reversals, and especially the degree of symmetry between partners in such turn-taking, that seem to be driving the brain and behavioral changes^39,43,48,49^. The focal rats of this study did not have more symmetrical play relationships with preferred partners compared to unpreferred partners. As play is used to familiarize unfamiliar individuals (e.g.,^48,49,70,71^), maintaining relationships with all members of the group may ensure that focal rats familiarize themselves with all individuals equally. An alternative explanation for why some rats do not form preferences, and why even the rats that do continue to play with all members of the groups may be that playing with multiple partners is rewarding and at the same time, it might be beneficial. By playing with a variety of partners, they are maximizing the variation of social experiences they gain. This might serve as a benefit to the orbitofrontal cortex which changes based on the number and novelty of partners^41,72^. Given that there is significant individual variation in play style and frequency, when given a choice, the present findings raise the possibility that rats switch from preferred to less preferred partners to maximize the benefits gained from playing.

Although males and female juvenile rats play using the same behavioral patterns^27^, and gain some of the same benefits from playing in the juvenile period^9^, females also gain some unique benefits^16^. Consequently, studies of play partner choice in group settings should be replicated with females, as their preferred social niche may differ to that of males, and so highlight different influences involved in partner preferences, if such preferences exist.

## Conclusions

Our results demonstrate that some juvenile male rats express partner preferences when playing in a group of unfamiliar individuals while others do not. Despite some rats forming preferences, focal rats continued to play with all the available partners throughout the 10-min play session. The focal rat’s favorite partner was the partner that in turn directed the most play towards the focal rat, suggesting that being able to form mutual relationships is an important criterion for forming preferences. However, none of the other possible mechanisms (e.g., weight and dominance asymmetry, play style, play quality) that we assessed account for how these preferences were formed. Despite living in groups in the wild^31^, the study of group play and partner preferences is an understudied feature of play in rats, despite laboratory rats serving as a major model of play and its associated neurobiology^8^. Group play should be employed more regularly in studying rat play as it may provide new insights into the mechanisms of social cognition, development, and individual variation^73^.

## Data Availability

The data that support the findings of this study are available from the corresponding author, J.R.H., upon reasonable request.
